# Neuroimmune Interactions in Schizophrenia: Focus on Vagus Nerve Stimulation and Activation of the Alpha-7 Nicotinic Acetylcholine Receptor

**DOI:** 10.3389/fimmu.2017.00618

**Published:** 2017-05-31

**Authors:** Fabiana Maria das Graças Corsi-Zuelli, Fernanda Brognara, Gustavo Fernando da Silva Quirino, Carlos Hiroji Hiroki, Rafael Sobrano Fais, Cristina Marta Del-Ben, Luis Ulloa, Helio Cesar Salgado, Alexandre Kanashiro, Camila Marcelino Loureiro

**Affiliations:** ^1^Department of Neuroscience and Behavior, Ribeirão Preto Medical School, University of São Paulo, São Paulo, Brazil; ^2^Department of Physiology, Ribeirão Preto Medical School, University of São Paulo, São Paulo, Brazil; ^3^Department of Cellular and Molecular Biology, Ribeirão Preto Medical School, University of São Paulo, São Paulo, Brazil; ^4^Department of Pharmacology, Ribeirão Preto Medical School, University of São Paulo, São Paulo, Brazil; ^5^Department of Surgery, Center of Immunology and Inflammation, Rutgers University-New Jersey Medical School, Newark, NJ, United States; ^6^Department of Physiological Sciences, Federal University of São Carlos, São Carlos, Brazil; ^7^Department of Internal Medicine, Ribeirão Preto Medical School, University of São Paulo, São Paulo, Brazil

**Keywords:** schizophrenia, immune system, cytokines, inflammation, microglia, vagus nerve stimulation, alpha-7 nicotinic acetylcholine receptor, cholinergic anti-inflammatory pathway

## Abstract

Schizophrenia is one of the most debilitating mental disorders and is aggravated by the lack of efficacious treatment. Although its etiology is unclear, epidemiological studies indicate that infection and inflammation during development induces behavioral, morphological, neurochemical, and cognitive impairments, increasing the risk of developing schizophrenia. The inflammatory hypothesis of schizophrenia is also supported by clinical studies demonstrating systemic inflammation and microglia activation in schizophrenic patients. Although elucidating the mechanism that induces this inflammatory profile remains a challenge, mounting evidence suggests that neuroimmune interactions may provide therapeutic advantages to control inflammation and hence schizophrenia. Recent studies have indicated that vagus nerve stimulation controls both peripheral and central inflammation *via* alpha-7 nicotinic acetylcholine receptor (α7nAChR). Other findings have indicated that vagal stimulation and α7nAChR-agonists can provide therapeutic advantages for neuropsychiatric disorders, such as depression and epilepsy. This review analyzes the latest results regarding: (I) the immune-to-brain pathogenesis of schizophrenia; (II) the regulation of inflammation by the autonomic nervous system in psychiatric disorders; and (III) the role of the vagus nerve and α7nAChR in schizophrenia.

## Introduction

Mental disorders are a major clinical and scientific challenge in modern medicine with an estimated prevalence of approximately 17% of the population (1, 2). Schizophrenia is one of the most debilitating psychotic disorders due to the lack of effective treatment (3, 4). Schizophrenia is a chronic psychiatric disorder characterized by faulty perception and withdrawal from reality. Schizophrenia symptomatology comprises positive (delusions, hallucinations), negative symptoms (social withdrawal, apathy) cognitive alterations, disorganized thinking, and psychomotor disturbances (2). The average life expectancy of schizophrenia patients is 10–25 years less than the normal population due to health problems and a higher suicide rate (5–7). Despite its significant social implications, schizophrenia is neglected worldwide (3, 4, 8).

Current treatments for schizophrenia are inefficacious, and there is an unmet clinical need for new and safe therapeutic strategies (9–12). Schizophrenia is usually treated with typical or atypical antipsychotics. Typical antipsychotics often induce significant psychomotor side effects. Atypical antipsychotics are the usual first-line treatment, although they are associated with metabolic syndrome and an increased cardiovascular risk of death (11, 12). An explanation for the inefficacious treatments is the insufficient knowledge about the etiology of schizophrenia. Both groups of antipsychotics are believed to be antagonists for dopamine receptors in the brain, and thus, previous studies mostly focused on the dopaminergic system (13). Although dopaminergic dysfunction contributes to schizophrenia, the mechanisms leading to this dysfunction are unknown. Recent studies demonstrate an abnormal inflammatory profile that can cause neurotransmission dysfunction in schizophrenia (14, 15). Early infections and other immune alterations during pregnancy and development can contribute to schizophrenia and other neurological disorders (16–19). These studies are contemporary with recent investigations demonstrating that vagal stimulation controls both central and peripheral inflammation (20–24) and that vagal stimulation can provide therapeutic advantages for neuropsychiatric disorders, such as depression and epilepsy (25, 26). However, little is known about the potential of this mechanism for treating schizophrenia (27). We reasoned that vagal stimulation may control inflammation and provide novel therapeutic advantages for schizophrenia. In this article, we evaluate this hypothesis by reviewing autonomic vagal dysfunction in psychiatric disorders and discussing the potential of vagal stimulation and alpha-7 cholinergic receptor (α7nAChR) agonists for treating schizophrenia.

## Immune-to-Brain Pathogenesis: From Homeostasis to Inflammation

Unregulated inflammation induced by infection or trauma results in excessive production of inflammatory cytokines, such as tumor necrosis factor (TNF), interferon-γ (IFN-γ), and interleukins (IL-1β, IL-6, etc.). These cytokines influence the homeostasis of several organs, as well as the central nervous system (CNS) (28).

Despite the traditional view of the brain as an immunologically privileged site, multiple studies have demonstrated that the CNS interacts with peripheral inflammatory cytokines through several pathways, described as follows (29). First, the humoral pathway: peripheral cytokines diffuse into the CNS through circumventricular organs and structures lacking the blood–brain barrier (BBB). Second, the cellular pathway: peripheral immune cells enter the CNS due to alterations in the BBB permeability and through the actions of chemoattractant mediators. Third, the microbiota–gut–brain axis: the microbiota–gut can transmit signals to the brain *via* the vagus nerve, immune mediators, and microbial metabolites, thereby altering neurotransmission in the CNS (30, 31). Fourth, the recently discovered central lymphatic pathway or the glymphatic system: mediated by functional lymphatic vessels in the CNS (32). In this pathway, extracellular fluids (the cerebrospinal fluid and interstitial fluid) draining from the brain parenchyma to the cervical and lumbar lymph nodes facilitate the traffic of antigens and immune cells affecting peripheral and central inflammation (33). Finally, the neural pathway: the afferent vagus nerve detects peripheral inflammatory cytokines (TNF, IL-1β, IL-6) and transmits signals to the nucleus tractus solitarius, and thereby to the hypothalamus (29, 34). All these pathways serve as immune-to-brain cross talk that facilitate central inflammation and behavioral changes.

## The Inflammatory Hypotheses of Schizophrenia

A balance between the pro- and anti-inflammatory cytokines is critical for proper brain development (35). Epidemiological studies indicate that infections during development increase the risk of schizophrenia in adulthood (36–39). These studies report an association between elevated maternal inflammatory cytokines levels (especially IL-8 and TNF) and risk of schizophrenia in adult offspring (16, 37). It has been observed in preclinical studies that maternal immune activation in rodents induces inflammatory cytokines (IL-1β, IL-6, TNF) and reduces anti-inflammatory cytokines (IL-10) in both the maternal fluids and in the fetal brain, inducing schizophrenia-like behaviors in the offspring (35, 40). Likewise, direct IL-6 inoculation into pregnant rodents also induces schizophrenia-like abnormalities in the offspring. This effect is prevented by neutralizing IL-6 antibodies, genetic depletion of the IL-6 gene (IL-6 knockout) (35), or overexpression of anti-inflammatory cytokines (IL-10) in the macrophages of pregnant dams (41).

Genetic studies have demonstrated the implications of immune-related genes in schizophrenia (42). A Danish cohort study reported a significant relationship between severe infections and the risk of schizophrenia. A previous history of autoimmune disorders increases the risk of schizophrenia by 36%. This risk of schizophrenia increases up to 60% in patients with a previous history of infection and hospitalization (19). Several clinical studies demonstrate a chronic low-grade inflammation in schizophrenia (43–46). Early studies suggested that this chronic low-grade inflammation may be due to chronically activated macrophages that fail to properly control T-lymphocytes in the so called “macrophage-T-lymphocyte hypothesis” (47). Thereafter, Schwarz et al. (48) suggested that psychotic patients have a T helper cells type 2-profile (Th2) characterized by increased Th2-produced IL-4 and decreased T helper cells type 1 (Th1)-produced IFN-γ (48). In contrast, a shift away from Th2-produced IL-4 and toward Th1-produced IFN-γ was later highlighted, suggesting the involvement of transforming growth factor (TGF)-β in the Th1/Th2 regulation of schizophrenia. Although contradictory, these hypotheses concur that an inflammatory imbalance is involved in schizophrenia (49).

Recent meta-analyses indicate that acute and chronically ill patients demonstrate a low-grade inflammatory profile that correlates with the clinical symptoms of schizophrenia (43, 45, 46) (Table 1). This inflammatory profile was also reported in drug-naïve patients in the first episode of psychosis (45). Since these patients were drug-naïve and in the first manifestation of the disease, it is unlikely that inflammation was related to antipsychotics or duration of illness. Thus, inflammatory cytokines in the peripheral blood were suggested to be either state or trait biomarkers. State biomarkers refer to specific cytokines elevated in schizophrenia and normalized with antipsychotics. Trait biomarkers are cytokines that are elevated in schizophrenia and are not normalized following antipsychotic treatment (43).

**Table 1:** **Cytokine profile in schizophrenia**.

**Meta-analyses (reference)**	**Number of studies included in the meta-analyses**	**Patients (status)**	**Peripheral blood cytokines**
Miller et al. (43)	33	AR	↑ IL-6, IL-8, TNF, IFN-γ, TGF-β, IL-1RA ↓ IL-10
		FEP (drug-näive)	↑ IL-1β, IL-6, IL-12, IFN-γ, TNF, TGF-β, sIL-2R
Upthegrove et al. (45)	14	FEP (drug-näive)	↑ IL-1β, sIL-2R, IL-6, TNF
Goldsmith et al. (46)	40	AR/FEP	↑ IFN-γ, IL-1β, IL-6, IL-8, IL-10^a^, IL-12, TNF, TGF-β, IL-1RA, sIL-2R ↓ IL-4, IL-10^a^
	18	Chronic	↑ IL-1β, IL-6, TNF, sIL-2R ↓ IFN-γ

*Enhanced (↑) or decreased (↓) cytokines levels in the peripheral blood of patients with schizophrenia; AR, acutely relapsed; FEP, first episode psychosis; IL, interleukin; TNF, tumor necrosis factor, IFN-γ, interferon-γ; TGF-β, transforming growth factor-β; sIL-2R, soluble IL-2 receptor; IL-1RA, IL-1 receptor antagonist*.

*^a^IL-10, increased in FEP but decreased in AR*.

The association between biological and environmental factors can have significant implications in schizophrenia (50). In this respect, Monji et al. (51) shed light on the microglia hypothesis of schizophrenia (51). Microglia are the resident macrophages in the CNS (52), and similar to peripheral macrophages, they show different activation states. Basal state microglia (M0) perform phagocytosis and promote neurite outgrowth (53–55). However, both physical (infections) (56) or psychological (early life stress) stressors induce microglial activation (57–62). In response to these events, microglial polarization is triggered, resulting in an inflammatory state (microglia type 1; M1) (63, 64). M1 microglia produce large amounts of inflammatory cytokines (TNF, IL-1, IL-6, IL-12) inducing neuronal cytotoxicity (57, 61, 62). In contrast, anti-inflammatory cytokines (IL-4, IL-10) induce microglial polarization toward an anti-inflammatory state (microglia type 2; M2), critical for homeostasis. The imbalance between these factors affects neurite outgrowth, neuronal connections, and neurotransmitter formation and induces neuronal cytotoxicity, contributing to neuropsychiatric disorders (57, 65–67). Indeed, increased microglial density and microglial activation have been demonstrated in the hippocampus and gray matter of schizophrenic patients, as demonstrated by postmortem and *in vivo* studies (68–73), and microglial activation has been linked to the pre-suicidal stress associated with schizophrenia (74).

Microglia-produced TNF induces neurotoxicity and neurodegeneration as demonstrated both *in vitro* (54, 75) and *in vivo* (76, 77). A typical example is that abnormal microglia activation alters tryptophan metabolism along the kynurenine pathway, producing metabolites that act as *N*-methyl-d-aspartate receptor (NMDAR)-agonists (quinolinic acid) or -antagonists, such as kynurenic acid (KYNA) (29, 78, 79). NMDAR dysfunction is associated with schizophrenia (80) and NMDAR-antagonists induce positive, negative, and cognitive symptoms in healthy volunteers, similar to those observed in schizophrenia (81, 82). Delusions and hallucinations related to autoantibodies blocking NMDARs were reported in schizophrenic and healthy controls (83, 84). The kynurenine pathway is also linked to oxidative stress. Neuronal apoptosis and structural changes in specific areas of the brain, such as the amygdala, hippocampus, and prefrontal cortex, are related to several psychiatric disorders, including schizophrenia (78). Together these studies demonstrate that inflammation of the CNS can contribute to schizophrenia (43, 45, 46).

The efficacy of antipsychotics may be due to microglial suppression and subsequent neuroprotection (85–87). Atypical antipsychotics inhibit TNF production by the IFN-γ-stimulated microglia (86, 87). Minocycline, a non-psychotic medication with potent effects in inhibiting microglia, has been suggested as an adjuvant in the treatment of schizophrenia (86). However, atypical antipsychotics induce metabolic and cardiovascular dysfunctions (11, 12). Thus, there is an unmet clinical need for new therapeutic strategies to control inflammation and the progression of schizophrenia.

## Does Autonomic Immunomodulation Contribute to the Inflammatory Component of Schizophrenia?

### The Autonomic Nervous System

The autonomic nervous system regulates the immune system through both the sympathetic and parasympathetic networks (21, 88, 89). This regulation is not only critical for physiological homeostasis, such as that in the gastrointestinal tract (90, 91), but also in pathological conditions that range from infection to trauma (22, 92–94). Briefly, sympathetic preganglionic neurons that originate from the thoracic and lumbar spinal segments synapse with postganglionic neurons in pre- or paravertebral ganglia. Parasympathetic preganglionic neurons originate from the brainstem and the sacral spinal cord and synapse with postganglionic neurons in terminal ganglia located near target organs. Both preganglionic sympathetic and parasympathetic neurons release acetylcholine (ACh). While all parasympathetic postganglionic neurons release ACh, most sympathetic postganglionic neurons release norepinephrine. Overall, sympathetic activity predominates during the “fight-or-flight” reactions, while parasympathetic activity predominates during “quiet” resting conditions (95). The vagus nerve—the major component of the parasympathetic system—plays a critical role in the communication between the brain and peripheral organs, such as the heart, lungs, and intestine (96).

### The Autonomic Nervous System Regulation of Inflammation in Schizophrenia

Dysfunction of the autonomic nervous system may contribute to the inflammatory profile reported in schizophrenia. The balance between the sympathetic and parasympathetic systems can be determined by the heart rate variability (HRV), which represents the variation of the intervals between heartbeats (97). Parasympathetic nerves slow heart rate and increase HRV by releasing ACh. Sympathetic nerves accelerate heart rate and decrease HRV by releasing epinephrine and norepinephrine (98, 99). Lower HRV is a predictor of cardiac morbidity and mortality (100–102). Psychiatric patients tend to have an autonomic imbalance with low HRV suggesting a reduced parasympathetic and increased sympathetic tone (103–105). Low HRV has also been related to psychotic symptoms and depression (106–108); and thus, the vagal tone could serve as an index of the treatment response (109).

The polyvagal theory associates the autonomic neuronal system with affective experiences and contingent social behavior (110). Low vagal activity is associated with reduced social involvement and a less flexible behavioral response to environmental conditions (110). In agreement with this theory, Bylsma and coworkers suggested that “*the cardiac autonomic balance may be a useful index that reflects the balance of the autonomic nervous system to respond to aspects of the environment that may be sensitive to psychophysiological abnormalities*” (111). Thus, autonomic neuronal dysfunction and low vagal activity could contribute to schizophrenia.

Electrical vagus nerve stimulation (VNS) was approved by the food and drug administration for treating several neuropsychiatric disorders including refractory epilepsy and depression (25, 112–114). However, few studies have explored VNS in schizophrenia (27). The only study that addresses VNS in schizophrenia examined the effects of transcutaneous vagal stimulation (tVNS) (115). tVNS is a non-invasive electrical stimulation of the external ear allowing stimulation of the auricular vagal branch (116). tVNS of the cymba conche results in the strongest activation of the vagal afferent pathway in the brainstem, as observed through functional magnetic resonance imaging (117). A bicentric, randomized, sham-controlled and double-blind clinical investigation was performed in 20 schizophrenic patients, who were randomly assigned to two groups: one received daily active stimulation of the left auricle for 26 weeks; the other group received sham stimulation daily. Regarding efficacy, there was no difference between the sham and tVNS groups (115). However, only half of the patients adhered to the protocol. Given that the vagal stimulation treatment depends on patient adherence, it was not possible to conclude a result due to non-adherence to the protocol and methodological limitations. In contrast, experimental studies demonstrated that VNS significantly reversed hippocampal hyperactivity, mesolimbic dopaminergic dysfunction, and schizophrenia-like symptoms, including cognitive deficits (118, 119).

Autonomic dysfunction facilitates immune alterations and increases the susceptibility to infectious and immunological disorders. The vagus nerve directs the “cholinergic anti-inflammatory pathway” modulating inflammation, as reported in preclinical and clinical studies (21, 23, 24, 26, 93, 118–124). In clinical studies, VNS inhibited cytokine production, improved HRV, and ameliorated low moods and emotional symptoms in depressive patients resistant to pharmacological treatment (26, 124). Recent studies demonstrated brain inflammation reduction with VNS applied at a low frequency, a protocol that favors the activation of efferent vagus nerve fibers (23, 24). Inhibition of CNS inflammation can be a consequence of peripheral inflammation inhibition (22, 24). The vagal anti-inflammatory signals are mediated by α7nAChR, suggesting that nicotinic agonists mimic vagal anti-inflammatory potential (22, 125, 126). α7nAChR were detected in several cell types, including neurons and immune cells. In the CNS, α7nAChR are expressed by pyramidal interneurons (127, 128), immature granule cells (129), astrocytes (130), and microglia (131, 132). In the periphery, this receptor is expressed in monocytes (133, 134), dendritic cells (135), macrophages (120, 136), T-cells (137), and B-cells (138). In this regard, the use of selective α7nAChR-agonists in the treatment of psychiatric and neurological patients has been reported (139). Remarkably, activation of α7nAChR in cultured microglia cells inhibits LPS-induced release of cytokines and promotes conversion of M1 microglia to M2 (132, 140).

Genetic studies demonstrated that α7nAChR activity is reduced, especially in the hippocampus, thalamus, frontal cortex, brainstem, ventral tegmental area, nucleus accumbens, and the cingulate cortex of schizophrenic patients (141–146). This reduced activity is more remarkable in gamma-aminobutyric acid (GABA) interneurons (142) that are key players in schizophrenia, especially in the cognitive domain (147). In addition, α7nAChR participate in NMDA and GABA_A_ receptors activity, and similar to NMDAR, they modulate calcium influx facilitating neurotransmission (148–150). Accordingly, α7nAChR has been involved in a myriad of brain functions, including learning, memory, neuroprotection, and synaptic plasticity (151–153). Conversely, α7nAChR dysfunction leads to abnormal NMDAR/GABA_A_ function and perturbation of glutamatergic and GABAergic neurotransmission (154).

Kynurenic acid, besides acting as an NMDAR inhibitor, is also a potent non-competitive α7nAChR-antagonist (155) and is associated with hypoglutamatergic and hypocholinergic neurotransmission, facilitating cognitive deficits and sensory gating disturbances in schizophrenia (155). α7nAChR-agonists restore dopamine signaling in the brain (156) and improve negative symptoms and cognitive function in schizophrenia (139, 157–161). Variation in brain KYNA may be related to the nicotinic cholinergic system. It has been observed that nicotine reduces levels of KYNA in clinical trials (162). In rodents, this effect was clear during a 5-day nicotine treatment; however, prolonged treatment enhanced central levels of KYNA (155). Notably, increased brain levels of KYNA are reported in schizophrenia (14); this concurs with data demonstrating a high rate of cigarette smokers with schizophrenia (163). For instance, over 80% of schizophrenic patients were smokers compared to 20% of the general population of the USA in 2006 (164). Accordingly, a recent meta-analysis reported that people who smoke are three times more likely to suffer psychosis (165); thus, high cigarette smoking in schizophrenia is suggested as a physiological basis on which patients try to correct cognitive deficits caused by α7nAChR dysfunction (155). In a recent study, chronic nicotine reversed hypofrontality in an animal model of addiction and schizophrenia (166). α7nAChR represents a potential therapeutic target for cognitive deficits and sensory gating disturbances; nevertheless, cigarette smoking is toxic and unspecific with deleterious side effects, and it is critical to find specific and safer therapeutic strategies for schizophrenia (163).

Essentially, the development of schizophrenia is more complex. This condition is influenced by genetic vulnerability interacting synergistically with multiple environmental risk factors, such as infections or stress in early life, drug abuse, besides other environmental adversities occurring at critical periods of neurodevelopment (167–169). This gene–environmental interaction could produce a latent immune vulnerability. Thus, when this vulnerability is manifested, the individuals become more susceptible to immune dysfunctions, increasing their risk of developing schizophrenia (170).

**Figure 1:**
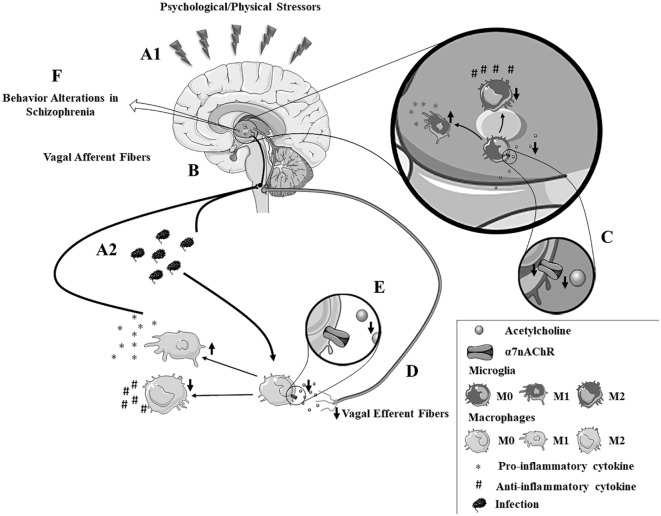
**The autonomic hypothesis of schizophrenia**. **(A1,A2)** Psychological or physical stressors contribute to enhanced production of inflammatory cytokines by both peripheral and brain immune cells. **(B)** The afferent vagus nerve facilitates immune-to-brain communication, by transmitting signals from the periphery to the brain. **(C)** Patients with schizophrenia have an intense autonomic imbalance characterized by α7nAChR dysfunction and reduced vagal tone. **(D)** The efferent vagus nerve plays a key role in the “cholinergic anti-inflammatory pathway,” a mechanism dependent on **(E)** acetylcholine binding to the α7nAChR, a pathway that is dysfunctional in schizophrenia patients. This impairment in the inflammatory reflex may contribute to **(F)** neuroinflammation and disrupted synthesis of neurotransmitters in schizophrenia. Note: the vagus nerve is constituted by both efferent and afferent fibers. The division shown in this figure is merely illustrative to explain the afferent and efferent neuroimmune routes.

Notably, stressful situations can induce an impairment of the α7nAChR (171–173). Animal models demonstrate an interaction between α7nAChR and the hypothalamic–pituitary–adrenal axis, a primary system responsible for the stress response (172). Prenatal restraint stress decreases α7nAChR expression in the hippocampus and prefrontal cortex in adult rats (173), while VNS reduces conditioned fear in rodents with posttraumatic stress disorder (174). A recent review stated that α7nAChR-agonists induce beneficial effects in patients with psychiatric disorders (139) ameliorating cognitive deficits, negative symptoms, and sensory gating disturbances in both preclinical and clinical trials of schizophrenia (139, 157–161). Advantageous effects for the negative symptoms have been reported repeatedly, while improvements in the cognitive domain remain controversial, deserving further exploration (175, 176). Together, these studies indicate that the vagus nerve and the α7nAChR may be involved in the inflammatory hypothesis of schizophrenia (Figure 1). Thus, future investigations are critical to determine their clinical potential in schizophrenia and other neurological disorders. Moreover, the consideration of stressful events in future investigations would be of interest. This would help to reduce the discrepancy regarding inflammatory processes in schizophrenia that are observed in data from several studies.

## Future Perspectives

The inter-relationship between the nervous and the immune systems is critical to understand the pathogenesis of schizophrenia. In brief, a reduced parasympathetic tone could contribute to inflammation observed in schizophrenic patients. This mechanism combined with stress-mediated dysfunctions of the α7nAChR can enhance the impairment of the inflammatory reflex, contributing to schizophrenia’s symptoms. In the face of microglial hyperactivation, future investigations controlling microglial activation through innovative approaches, such as VNS and α7nAChR modulation, may provide clinical advantages for treating schizophrenia. As early exposure to stressors induces changes in the inflammatory reflex, a better understanding of the association between biological and environmental factors would potentially improve the diagnosis and treatment of schizophrenia. In this regard, public health interventions controlling stressful events, such as public education and comprehensive approaches to early treatment focusing on individual, social and environmental factors, might be beneficial for mental health promotion and prevention of future psychiatric disorders.

## Author Contributions

AK proposed the review to the authors and together with HS, LU, and CD-B revised the manuscript. FC-Z and CL suggested the topic for this review, coordinated the research group, drafted and revised this manuscript. FB also drafted and revised the manuscript, and together with AK, HS, and LU was essential in the consideration of the autonomic nervous system. GQ and CH were helpful in providing general information about inflammation. RF participated in the elaboration of the figure. All authors approved the final manuscript.

## Conflict of Interest Statement

The authors declare that the research was conducted in the absence of any commercial or financial relationships that could be construed as a potential conflict of interest.

## Abbreviations

ACh, acetylcholine; BBB, blood–brain barrier; CNS, central nervous system; GABA, gamma-aminobutyric acid; HRV, heart rate variability; IL, interleukin; IFN, interferon; KYNA, kynurenic acid; NMDAR, *N*-methyl-d-aspartate receptor; M1, microglia type 1; M2, microglia type 2; TGF, transforming growth factor; Th1, T helper cells type 1; Th2, T helper cells type 2; TNF, tumor necrosis factor; VNS, vagus nerve stimulation; tVNS, transcutaneous vagus nerve stimulation; α7nAChR, alpha-7 nicotinic acetylcholine receptor.
